# Transcription analysis on response of swine lung to H1N1 swine influenza virus

**DOI:** 10.1186/1471-2164-12-398

**Published:** 2011-08-08

**Authors:** Yongtao Li, Hongbo Zhou, Zhibin Wen, Shujuan Wu, Canhui Huang, Guangmin Jia, Huanchun Chen, Meilin Jin

**Affiliations:** 1 Unit of Animal Infectious Diseases, State Key Laboratory of Agricultural Microbiology, Huazhong Agricultural University. 1 Shizishan Street, Wuhan, Hubei, 430070, P.R. China; 2 College of Veterinary Medicine, Huazhong Agricultural University, P.R. China. Shizishan Street, Wuhan, Hubei, 430070, P.R. China

## Abstract

**Background:**

As a mild, highly contagious, respiratory disease, swine influenza always damages the innate immune systems, and increases susceptibility to secondary infections which results in considerable morbidity and mortality in pigs. Nevertheless, the systematical host response of pigs to swine influenza virus infection remains largely unknown. To explore it, a time-course gene expression profiling was performed for comprehensive analysis of the global host response induced by H1N1 swine influenza virus in pigs.

**Results:**

At the early stage of H1N1 swine virus infection, pigs were suffering mild respiratory symptoms and pathological changes. A total of 268 porcine genes showing differential expression (DE) after inoculation were identified to compare with the controls on day 3 post infection (PID) (Fold change ≥ 2, p < 0.05). The DE genes were involved in many vital functional classes, mainly including signal transduction, immune response, inflammatory response, cell adhesion and cell-cell signalling. Noticeably, the genes associated with immune and inflammatory response showed highly overexpressed. Through the pathway analysis, the significant pathways mainly concerned with Cell adhesion molecules, Cytokine-cytokine receptor interaction, Toll-like receptor signaling pathway and MAPK signaling pathway, suggesting that the host took different strategies to activate these pathways so as to prevent virus infections at the early stage. However, on PID 7, the predominant function classes of DE genes included signal transduction, metabolism, transcription, development and transport. Furthermore, the most significant pathways switched to PPAR signaling pathway and complement and coagulation cascades, showing that the host might start to repair excessive tissue damage by anti-inflammatory functions. These results on PID 7 demonstrated beneficial turnover for host to prevent excessive inflammatory damage and recover the normal state by activating these clusters of genes.

**Conclusions:**

This study shows how the target organ responds to H1N1 swine influenza virus infection in pigs. The observed gene expression profile could help to screen the potential host agents for reducing the prevalence of swine influenza virus and further understand the molecular pathogenesis associated with H1N1 infection in pigs.

## Background

Swine influenza is a respiratory disease in pigs characterized by fever, anorexia, tachypnea, dyspnea and coughing [1]. The causative agent, swine influenza virus (SwIV) contains three key subtypes: H1N1, H3N2 and H1N2 circulating in pigs worldwide [2-5]. In pig flocks, H1N1, as the predominant subtype in clinical surveys or in the NCBI Influenza resource, has been further categorized into classical swine H1N1 virus, human-like H1N1 virus and European avian-like H1N1 virus [6-8]. In March 2009, a novel swine-origin H1N1 influenza virus containing a unique combination of gene segments of the North American and Eurasian SwIV lineages has continued to circulate in humans and raised severe concerns about pandemic developments. And the finding indicates the significant role of H1N1-subtype SwIV in the evolution of new viruses with pandemic potential [9].

SwIV replication is mainly restricted to epithelial cells in the respiratory tract with the lung being the major target organ [10]. As a short-lasting disease, swine influenza manifests itself with an incubation period of 1-3 days, then the recovery follows, which is limited to 6 or 7 days after infection [11]. During the acute phase of the disease, SwIV induces an overwhelming and simultaneous pro-inflammatory cytokines in the lungs of infected animals [12]. Recently, researchers have found that after H1N1 SwIV infection, secretion of innate, pro-inflammatory, Th1, Th2, and Th3 cytokines was observed in infected pig lungs, which could be useful indicator for host anti-viral response and SwIV pathogenesis [13]. Among the cytokines, several have been demonstrated to be related with anti-viral functions or tissue damage at the acute stage of SwIV infection, such as IFN-alpha [14], tumour necrosis factor-alpha [15], interleukins [16]. As mentioned above, swine influenza infection has the characteristics of lasting for a short period and quick recovery, which suggests that great number anti-viral molecules, such as IFN-stimulated genes, may play a central role in the infection course, although the detailed information remains to be obtained. Moreover, cytokines are suggested to be able to partly explain why invasive SwIV could cause severe tissue damage by influx of inflammatory cells, which makes animals more susceptible to secondary bacterial infections. In the clinical surveillance, SwIV was a frequent co-infecting agent in swine pneumonia cases [17]. In the previous studies, the experimental dual infection in pigs with a H1N1 SwIV and other pathogens were carried out, and the results determined the contributions of SwIV infection to the enhanced susceptibility to secondary bacteria induced pneumonia [18,19]. To date, investigators have mainly concentrated their attention on the implications of swine influenza for public health. However, it is equally worth elucidating basic viral pathogenesis of swine influenza, for swine influenza is one of main immunesuppressive diseases in pig flocks and causes the serious harm to the swine industry. Along this line, it is also indispensable to study the host response against SwIV infection and analyze the potential pathogenesis of SwIV infection.

Moreover, virus infections are generally associated with numerous changes in gene expression of a specific tissue or organ that determines the fate of the ultimate outcome of the infected host. As a high density technology, microarray gene expression profiling has been increasingly used to evaluate the status of gene expressions of pigs after being infected by different pathogens [20,21]. In the analysis of the gene regulation patterns, attention has been given to genes related to the key factor in the clinical course and pathology of the disease, particularly when the host was infected with zoonosis, like SwIV [22].

To obtain the sufficient information on host response of H1N1 SwIV infection in pigs, the present study focused on the global genomic analysis of transcriptional responses of pig lungs to H1N1 virus infection using Agilent Porcine Oligo Microarray containing more than 42,034 transcripts. The comparison of H1N1 SwIV-infected and mock-infected pig lungs indicated that 268 and 214 DE genes were differently expressed respectively on PID 3 and PID 7 (Fold change ≥ 2, p < 0.05). As a result, these data would enable us to better understand the underlying pathogenesis of H1N1 SwIV infection in pigs.

## Methods

### Animals and viruses

A total of 30 piglets with high-health status were obtained from a commercial herd and were raised in isolated facilities. To confirm that all piglets were free of swine influenza virus prior to challenge, Hemagglutination Inhibition Test (HI) and Enzyme-linked immunosorbent assay (ELISA) were performed on all pigs' sera and the sera were confirmed negative for the antibodies against H1 and H3 swine influenza viruses (unpublished data). The influenza viruses were stored at -80°C. The biological properties of swine influenza strains including EID_50 _and TCID_50 _were also determined (unpublished data). The representative of these strains, designated as A/swine/Hubei/101/2009(H1N1) was sequenced and submitted into the Genbank (Accession numbers: CY083005, CY083006, CY083007, CY083008, CY083009, CY083010, CY083011, CY083012).

### Virus infection and tissue collection

All procedures for animal infection and tissue collection were performed according to protocols approved by Biological Studies Animal Care and Use Committee in Hubei Province, PR China. At the age of day 35, 15 pigs were randomly allocated to the non-infected group and another 15 to the infected group. Each piglet in the infected group was intranasaly challenged with A/swine/Hubei/101/2009(H1N1) strain at a dose of 10^7.0^EID_50 _in 2 ml of phosphate buffered saline (PBS) in separate HEPA filtered containment facilities. Each piglet of the non-infected group was treated similarly with an identical volume of PBS as control. Clinical symptoms, including lethargy, anorexia, coughing, hyperpnea or dyspnea, nasal and ocular discharge, and their body temperature were recorded daily. 7 from the infected pigs and 7 from the control pigs were respectively euthanized on PID 3 and 7 by intravenous administration of pentobarbital, and their lungs were removed completely. The percentage of the surface of each pulmonary lobe affected by macroscopic lesions was estimated visually. Virus isolation and H1N1 specific quantitative real-time PCR (qRT-PCR) were performed after the pigs were sacrificed on PID 3 and 7. Quantification was achieved by relating viral Ct value to the Ct value on a standard curve of a measured copy number of a plasmid bearing a 140 bps fragment of the NP gene, amplified by using the following primers: 5-CACTCACCTGATGATTTGGCA-3 (forward) and 5- CAGCAGCTCCAGATCTCCTTG-3 (reverse). Some lungs from both groups were frozen and stored in liquid nitrogen immediately after collection until RNA extraction, and the remaining were fixed in formalin for further histopathology evaluation.

### RNA extraction, reverse transcription, RNA labelling and cRNA hybridization Total

RNA extraction from the lungs prepared from the same lesion, areas of localized infection (with viral mRNA present) was performed using TRIzol by following the standard instructions (Invitrogen, Carlsbad, CA) and a clean-up was carried out using RNeasy columns (Qiagen, Valencia, CA). Both RNA integrity and concentration were evaluated by Agilent 2100 Bioanalyzer by following the manufacturer's instructions (Agilent Technologies, Palo Alto, CA). Reverse transcription of 2 μg total RNA and synthesis of Cy3-labelled cRNA with one round of amplification were carried out by a commercial Agilent array service (Shanghaibio, China) following the standard one-cycle protocol according to the manufacturer's instructions. Transcriptional profiles were assessed using Agilent 4 × 44 K Porcine Oligo Microarray which contains more than 42,034 transcripts of pig from the database of RefSeq, Unigene and TIGR. Hybridization and scanning of the arrays were carried out according to standard protocols using a G2565BA Scanner (Agilent Technologies, Palo Alto, CA).

### Expression microarray analysis and bioinformatics

Raw data and statistical analyses were performed with Feature Extraction software. Normalization was performed per chip (normalized to 50th percentile) and per gene (normalized to the median) respectively. A statistical analysis of variance (ANOVA) model was applied to the data and the significance was showed by accepting a false discovery rate (FDR) of 0.05. A further cut-off threshold was applied based on a fold change of 2.0 between infected and control pigs. Then all the DE genes were performed for hierarchical cluster (Ver.3.0) and TreeView (Ver.1.1.1) analyses. Genes with significant similarities to the transcripts in nr database based on BLASTX searches were selected for GO analysis, performed by MAS 3.0 software which was based on DAVID database (CapitalBio, Beijing, China) [23]. Annotation results were obtained by inputting the list of gene symbol as identifier. The Pathway analysis was done using the MAS 3.0 software which was based on the Kyoto Encyclopedia of Genes and Genomes database (KEGG) (CapitalBio, Beijing, China). The raw and processed data discussed in this publication have been deposited in NCBI's Gene Expression Omnibus and are accessible through GEO Series accession number GSE28871.

### qRT-PCR for confirmation

Microarray results were validated by qRT-PCR using SYBR green-based detection with an ABI PRISM 7500 cycler (Applied Biosystems, Foster City, CA). Total lung RNA (1 μg) prepared as described above was reversely transcribed in a 20 ul reaction mixture containing 2ul avian myeloblastosis virus (AMV) buffer, 50 pM Oligo18T, 0.5 mM dNTPs, 10 U RNase inhibitor and 20 U AMV reverse transcriptase (TAKARA, Japan). All primers were designed using Primer Premier 5.0 software based on the sequences of the corresponding porcine mRNAs in GenBank (Additional file 1). The GAPDH gene was used for expression normalization, using the primers described previously [23]. PCRs were triplicated to guarantee the reproducibility of amplification of the lung cDNA sample from each animal. Changes in gene expression revealed by qRT-PCR were calculated by following the t-test reported previously [24], and a P-value < 0.05 was considered significant. Reaction specificity was ascertained by performing the Melt procedure at the end of the amplification protocol; and the efficiency of the PCR reaction was 92-99% for all reactions (slope standard line between-3.3 and-3.6) according to the manufacturer's instructions (Applied Biosystems, Foster City, CA).

## Results

### Clinical and Pathological evaluation

**Figure 1:**
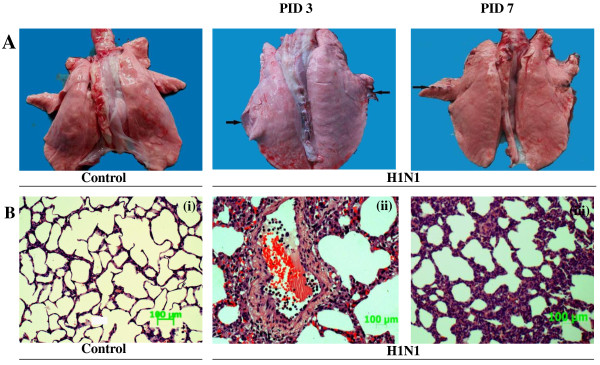
**The macroscopic and microscopic lung lesions in pigs infected with H1N1 SwIV**. (A) Pigs were PBS-mock infected or infected with H1N1 SwIV. On PID 3 and 7, extensive areas of macroscopic lesions of lungs tissues were observed in lungs of virus-infected pigs. (B) Hematoxylin- and eosin-stained sections of lungs from control pigs and the pigs infected with H1N1 SwIV. The microscopic lesions of lung tissues collected on PID 3 and 7 showed acute pneumonia with pathological changes: alveolar wall thickening, bleeding, debris in the lumen, and the accumulation of inflammatory cells, indicated by an arrow (panel ii and iii). The lungs of PBS-mock infected pigs showed normal bronchial epithelial lining and absence of inflammatory cells (panel i).

By monitoring the clinical signs of all pigs, it was intriguing to find that most infected pigs just showed mild signs, such as coughing, nasal discharge and dyspnea on PID 1-3 and recovered on PID 5-7. The average body temperature of infected pigs started to increase on PID 1, peaked around PID 3 with 40.8°C, and returned to the initial temperature (about 39°C) until PID 5 (P < 0. 05). As expected, the temperature of the six PBS-mock infected pigs rose slightly on PID 1 and kept stable all the remaining days. To investigate pathological damage to lungs of infected pigs, 7 from the infected pigs and 7 from the control pigs were respectively euthanized on PID 3 and 7 by intravenous administration of pentobarbital, and the macroscopic lesions of lungs tissues were estimated visually (Figure 1A). Then lungs of pigs in each group were fixed by submerging them in 4% neutral buffered formalin and embedding them in paraffin. Three-micron sections were made before they were stained with hematoxylin and eosin (H&E). Lung tissues in lesion areas of PID 3 showed dominant pneumonia with increased lung elastance, severe edema formation, and pathological responses, such as large amount infiltration of inflammatory cells, alveolar wall thickening and bleeding. The pathological changes of lung tissues on PID 7 were investigated, they just showed slightly alveolar wall thickening with few inflammatory cells, and the PBS-mock infected pigs didn't show lung lesions (Figure 1B).

### Virus isolation and confirmation

The virus could be isolated from lung tissues in all infected pigs on PID 3 and PID 7 but could only be found in nasal swabs of pigs before PID 3. As expected, no virus could be isolated in the controls. To investigate the tissue distribution of virus, samples of heart, liver, spleen, lung, kidney, trachea, tonsil, intestine and serum from pigs were collected on PID 3 and 7 after inoculation, then detected to manifest whether any virus could be isolated from these tissues. Results showed that in most infected pigs, the virus could be isolated only from the trachea and lung. No virus was isolated from heart, liver, intestine, spleen, tonsil or kidney in any infected pigs. The above results were confirmed by qRT-PCR using specific primers for the NP gene (unpublished data). Based on these results, we chose three pigs from each group to perform the following studies.

### Gene expression alterations in the lung tissue

**Figure 2:**
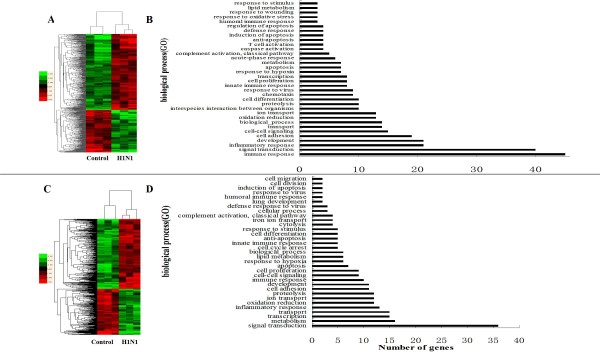
**Clustering and characterization of the differential expression of genes**. (A) The DE genes showing clear functional annotation on PID 3 have been selected for cluster analysis which is described in methods. Amongst the DE genes on PID 3, a set of 160 genes were up-regulated and the rest of 108 genes were down-regulated. Each row represents a separate gene and each column represents a separate piglet. Color legend is on the left, the color scale ranges from saturated green for log ratios -3.0 and above to saturated red for log ratios 3.0 and above. Red indicates increased gene expression levels; green indicates decreased levels compared with normal samples. (B) Categories of annotated genes based on biological process GO term. On PID 3, the DE genes mainly clustered into 34 functional groups with varied numbers. (C) The genes showing clear functional annotation on PID 7 have been selected for cluster analysis. (D) On PID 7, the DE genes mainly clustered into 33 functional groups with varied numbers. Many categories shared the same genes.

To analyse the genomic expression of SwIV-infected pigs, total RNA was extracted from the infected pig lungs (with viral mRNA present) and from the control pig lungs at the same area. The transcriptome analysis indicated that the detection ratios of all probe sets in the chip representing "expression transcripts" were 75-85% in different samples, which could exactly meet the quality control standards. The global expression profile of porcine lungs of the infected group was compared with that of the control group. After quantile normalization and statistical analyses, a total of 534 and 467 transcripts at acute and recovery phases were respectively identified to be differentially expressed (Fold change ≥ 2, p < 0.05). The functions of the DE genes were analysed by MAS 3.0 software. Surprisingly, about half of the transcripts at both time points were not functionally annotated, with only a total of 268 genes showing clear functional annotation on PID 3 and 214 genes on PID 7, and all DE genes were performed for hierarchical cluster (Figure 2A and 2C). Amongst the DE genes on PID 3, a set of 160 genes were up-regulated and the rest of 108 genes were down-regulated. The DE genes mainly clustered into functional groups: signal transduction (e.g. *TLRs, RLRs*), immune response (e.g. *GBP1, OAS2*), inflammatory response (e.g. *CXCL9, CXCL10*), cell adhesion (e.g. *SELL, SELE*), cell-cell signaling (e.g. *CCR5, TGF-β2*), biological process (e.g. *RETN, APOBEC3A*), transport (e.g. *Haptocorrin, AQP7*), oxidation reduction (e.g. *DUOX2, ALOX15*), interspecies interaction between organisms (e.g. *IFIH1, STAT1*), response to virus (e.g. *MX1, ISG20*) and so on (Figure 2B). Particularly, the genes associated with immune and inflammatory response were highly overexpressed among the up-regulated genes, which indicated that they may play important roles in host defense response at the early stage of infection (Table 1). Through similar analysis, on PID 7, it was found that the predominant groups of DE genes contained signal transduction, metabolism, transcription, development and transport, however, the DE genes related with immune and inflammatory responses became the minority in number (Table 2). The results presented here reflected that it was beneficial for the host to prevent excessive inflammatory damage and recover the normal state by activating these clusters of genes mentioned above (Figure 2D). When comparing the DE genes on PID 3 and 7, only 23 genes were present in the two time points, and they may exert a continuous impact on host at both early and recovery stages (Additional file 2). The complete microarray dataset of the two time points with functional annotations and the changes at expression level is summarized in Supplementary Table (Additional file 3).

**Table 1:** The DE genes associated with immune and inflammatory responses on PID 3

Functional classification	Gene description	Gene symbol	Fold Change	Gen Bank ID
	CD45 antigen isoform 3 Fragment	CD45	38.62459	AK230523
	Sus scrofa antibacterial protein	PMAP-23	21.70289	EW419136
	Sus scrofa peptide antibiotic PR39	PR39	13.50724	NM_214450
**immune**	Sus scrofa chemokine ligand 26-like mRNA	CCL26	8.645769	NM_001078665
**response**	Sus scrofa protegrin 4 NPG4, mRNA	NPG4	8.237142	NM_213863
	Sus scrofa immunoglobulin kappa variable group	IGKV	6.440473	CB286369
	Sus scrofa 2'-5'-oligoadenylate synthetase-like mRNA	OASL	5.803129	NM_001031790
	Interferon-stimulated gene 20 kDa protein	ISG20	5.414398	NM_001005351.2
	Sus scrofa chemokine C-X-C motif ligand 10 mRNA	CXCL10	5.329808	NM_001008691
	*Rep: IL-1 receptor 2-Bos taurus Bovine, partial 98%*	IL1R2	5.321422	NM_001046210.1
	Sus scrofa MHC class II histocompatibility antigen	SLA-DQA1	5.232868	AK230483
	Sus scrofa cathepsin L1-like	CTSL1	4.814094	AK230898
	Sus scrofa interferon regulatory factor 3 (IRF3), mRNA	IRF3	4.320395	NM_213770
	Sus scrofa Fc fragment of IgG, low affinity IIb, receptor, mRNA	FCGR2B	3.622257	NM_001033013
	Sus scrofa CD14 molecule, mRNA	CD14	3.544918	NM_001097445
	Sus scrofa interferon stimulated gene 15 mRNA	ISG15	3.45	EU584557.1
	Sus scrofa IgG heavy chain LOC396781, mRNA	IgG	3.438582	NM_213828
	Sus scrofa interferon-induced protein with tetratricopeptide repeats 1	IFI56	3.386147	XM_001928724
	Sus scrofa inducible T-cell co-stimulator ICOS, mRNA	ICOS	3.353116	NM_001044546
	Sus scrofa interferon regulatory factor 7 IRF7, mRNA	IRF7	3.249327	NM_001097428
	Sus scrofa myxovirus influenza virus resistance 1, interferon-inducible protein p78 MX1, mRNA	MX1	3.237013	NM_214061
	Sus scrofa MHC class I antigen	SLA-2	3.187732	AK231553
	Sus scrofa cytotoxic T-lymphocyte-associated protein 4 CTLA4, mRNA	CTLA4	3.184663	NM_214149
	Sus scrofa DEAD Asp-Glu-Ala-Asp box polypeptide 58 DDX58, mRNA	DDX58	3.122048	NM_213804
	*Rep:Tumor necrosis factor receptor superfamily member 17-Homo sapiens*	TNFRSF17	3.071191	NM_001192.2
	Sus scrofa CD8a molecule CD8A, mRNA	CD8A	2.834259	NM_001001907
	Similar to melanoma cell adhesion molecule	CD146	-2.819245	BX915975
	*similar to guanylate binding protein 4*	GBP4	2.780124	AK239735
	Sus scrofa guanylate binding protein 1, interferon-inducible, 67 kDa GBP1, mRNA	GBP1	2.765026	NM_001128473
	Sus scrofa ubiquitin specific peptidase 18, mRNA	USP18	2.732302	NM_213826
	Sus scrofa interferon-induced protein with tetratricopeptide repeats 2 mRNA	IFI54	2.729199	AK230663
	*mast cell immunoreceptor signal transducer-like*	CLNK	2.704822	AK237804
	*Rep:CD274 molecule CD274, mRNA- Homo sapiens*	CD274	2.648947	EW655654
	Sus scrofa CD163 molecule CD163, mRNA	CD163	2.633336	NM_213976
	Sus scrofa interferon-induced protein 44 mRNA	IFI44	2.629777	AK233687
	Sus scrofa interleukin 12A natural killer cell stimulatory factor 1, IL12A, mRNA	IL12A	2.580563	NM_213993
	Sus scrofa guanylate binding protein 2, interferon-inducible GBP2, mRNA	GBP2	2.561492	NM_001128474
	Sus scrofa interferon-induced protein with tetratricopeptide repeats 3, mRNA	IFI60	2.465327	XM_001928703
	Sus scrofa interferon-induced transmembrane protein 1	IFITM1	2.407903	AK237820
	Sus scrofa chemokine C-C motif receptor 5, mRNA	CCR5	2.374719	NM_001001618
	Sus scrofa chemokine C-C motif receptor 10, mRNA	CCR10	2.36215	NM_001044563
	Sus scrofa chemokine C-C motif ligand 28, mRNA	CCL28	2.330211	NM_001024695
	Sus scrofa chemokine C-X-C motif ligand 9 mRNA	CXCL9	2.312722	BP169836
	Sus scrofa transporter 1, ATP-binding cassette, sub-family B MDR/TAP TAP1, mRNA	TAP1	2.15635	NM_001044581
	Sus scrofa Fas ligand TNF superfamily, member 6, mRNA	FAS	2.098484	NM_213806
	Sus scrofa immunoglobulin-associated alpha CD79A, mRNA	CD79A	2.086904	NM_001135962
	Sus scrofa interferon induced with helicase C domain 1 IFIH1, mRNA	IFIH1	2.075703	NM_001100194
	Sus scrofa aquaporin 9, mRNA	AQP9	2.067154	NM_001112684
	Sus scrofa tumor necrosis factor superfamily member 18 mRNA, partial cds	TNFSF18	2.065184	DQ995649
	Sus scrofa 2'-5'-oligoadenylate synthetase 2, 69/71kDa OAS2, mRNA	OAS2	2.054377	NM_001031796
	*similar to cystatin F*	CST7	2.042642	CJ009409
	Sus scrofa Ig gamma 2b chain constant region	IGG2B	2.017483	AK237225
	Sus scrofa CD86 molecule CD86, mRNA	CD86	2.007655	NM_214222
	Sus scrofa vitronectin VTN, mRNA	VTN	-2.006228	NM_214104
	Sus scrofa chemokine ligand 26-like protein LOC780409, mRNA	CCL26	8.645769	NM_001078665
	Sus scrofa Toll-like receptor 3	TLR3	7.331916	NM_001097444.1
	Sus scrofa chemokine C-X-C motif ligand 10, mRNA	CXCL10	5.329808	NM_001008691
	Sus scrofa selectin L (SELL), mRNA	SELL	4.497706	NM_001112678
**inflammatory**	Sus scrofa inflammatory response protein 6, mRNA	IRG6	3.840661	NM_213817
**response**	Sus scrofa C1q-related factor LOC780424, mRNA	CRF	3.627351	NM_001078676
	Sus scrofa CD14 molecule CD14, mRNA	CD14	3.544918	NM_001097445
	Sus scrofa alpha-1-antichymotrypsin 2 SERPINA3-2	SERPINA3	3.119463	NM_213787
	Sus scrofa complement C4 C4, mRNA	C4	2.949702	NM_001123089
	Sus scrofa selectin E SELE, mRNA	SELE	-2.879741	NM_214268
	Sus scrofa toll-like receptor 2 (TLR2), mRNA	TLR2	2.8723	NM_213761
	Similar to melanoma cell adhesion molecule	CD146	-2.819245	BX915975
	Sus scrofa toll-like receptor 8 TLR8, mRNA	TLR8	2.675719	NM_214187
	Sus scrofa CD163 molecule CD163, mRNA	CD163	2.633336	NM_213976
	*Rep: transforming growth factor, beta 2- Macaca mulatta*	TGFB2	-2.554227	EV858461
	Sus scrofa chemokine C-C motif receptor 5, mRNA	CCR5	2.374719	NM_001001618
	Sus scrofa chemokine C-C motif ligand 28, mRNA	CCL28	2.330211	NM_001024695
	Sus scrofa chemokine C-X-C motif ligand 9	CXCL9	2.312722	BP169836
	Sus scrofa serpin peptidase inhibitor, clade E member 1 SERPINE1, mRNA	SERPINE1	2.225002	NM_213910
	Sus scrofa Toll-like receptor 9 TLR9, mRNA	TLR9	2.200666	NM_213958
	*Rep: arachidonate 15-lipoxygenase Alox15 gene- Mus musculus*	Alox15	-2.168114	AK238353
	Sus scrofa signal transducer and activator of transcription 1 STAT1, mRNA	STAT1	2.116864	NM_213769
	Sus scrofa S100 calcium binding protein A2	S100A2	2.04835	XM_001929556
	Sus scrofa serpin peptidase inhibitor, clade G1 inhibitor, member 1 SERPING1, mRNA	SERPING1	2.022489	NM_001123194

The DE genes associated with immune and inflammatory responses were assigned based on GO term and manual annotation. Manual annotations were listed in italics. Most genes could also be classified into other categories but only immune and inflammatory responses were considered.

**Table 2:** The DE genes associated with immune and inflammatory responses on PID 7

Functional classification	Gene description	Gene symbol	Fold Change	Gen Bank ID
	*Rep:CD274 molecule CD274, mRNA- Homo sapiens*	CD274	2.648947	EW655654
**immune**	Sus scrofa complement component C6	C6	2.660923	NM_001097449
**response**	Sus scrofa lymphocyte cytosolic protein 2	LCP2	2.461111	AK230876
	*Rep:Sus scrofa tumor necrosis factor superfamily member 18 mRNA*	TNFSF18	2.5221772	DQ995649
	Sus scrofa interleukin 2 receptor, alpha	IL2RA	3.3108058	NM_213835
	Sus scrofa C-type lectin domain family 5, member A	CLEC5A	4.702034	NM_213990
	Sus scrofa interferon stimulited exonuclease gene 20 kDa	ISG20	2.1768405	NM_001005351
	Sus scrofa MHC class II DR-alpha (SLA-DRA)	SLA-DRA	2.0392556	NM_001113706.1
	Sus scrofa secreted phosphoprotein 1	SPP1	3.411632	NM_214023
	Sus scrofa lymphocyte-activation gene 3	LAG3	2.0111256	NM_001105306

	Sus scrofa selectin E	SELE	9.232597	NM_214268
	Sus scrofa chemokine ligand 26-like mRNA	CCL26	8.645769	NM_001078665
	Sus scrofa Toll-like receptor 3	TLR3	7.331916	NM_001097444.1
	Sus scrofa matrix metallopeptidase 25	MMP25	3.32788	NM_213905
	Sus scrofa toll-like receptor 7	TLR7	3.262197	NM_001097434
**inflammatory response**	Sus scrofa interleukin 13	IL13	2.4685268	NM_213803
	Sus scrofa sphingosine 1-phosphate receptor 3-like	S1PR3	2.293045	BP173206
	chemokine (C-C motif) ligand 8	CCL8	2.0915823	NM_001164515.1
	Sus scrofa S100 calcium binding protein A2	S100A2	2.04835	XM_001929556
	Sus scrofa Toll-like receptor 5	TLR5	- 2.10299	NM_001123202
	*Rep: transforming growth factor, beta 2- Macaca mulatta*	TGFB2	- 2.180843	EV858461
	*Rep: leukocyte cell-derived chemotaxin 2*	LECT2	- 2.381497	NM_002302.2
	Sus scrofa S100 calcium binding protein A16	S100A16	- 13.31639	NM_001190208.1

The DE genes associated with inflammatory response were assigned based on GO term and manual annotation. Manual annotations were listed in italics. Most genes could also be classified into other categories but only immune and inflammatory responses were considered.

### Pathway analysis

To gain insight into the different biological processes of swine influenza infection at the different time points, a pathway analysis was performed on DE genes. We selected most genes for further examination, based on their potential implication in immune and inflammatory response processes. On PID 3, by using the KEGG database, the significant pathways mainly contained Cell adhesion molecules, Cytokine-cytokine receptor interaction, Toll-like receptor signaling pathway and MAPK signaling pathway, which suggested that at the early stage, the host took different strategies to activate immune and inflammatory response so as to prevent from virus infections. However, on PID 7, PPAR signaling pathway and Complement and coagulation cascades became the most significant pathways, indicating that the host start to repair excessive tissue damage by anti-inflammatory functions at the recovery stage (Table 3).

**Table 3:** The key pathways that contain an over-representation of regulated genes in pig lungs after incubation with H1N1 SwIV on PID 3 and 7

KEGG pathway(PID 3)	Gene number	KEGG pathway(PID 7)	Gene number
Cell adhesion molecules (CAMs)	17	PPAR signaling pathway	8
Cytokine-cytokine receptor interaction	15	Focal adhesion	8
Toll-like receptor signaling pathway	13	Cytokine-cytokine receptor interaction	8
MAPK signaling pathway	13	Complement and coagulation cascades	7
Focal adhesion	12	Wnt signaling pathway	6
Complement and coagulation cascades	10	Adherens junction	4
T cell receptor signaling pathway	8	ECM-receptor interaction	4
Antigen processing and presentation	7	Toll-like receptor signaling pathway	4
PPAR signaling pathway	5	Cell cycle	4
ECM-receptor interaction	5	Cell adhesion molecules (CAMs)	4
Jak-STAT signaling pathway	4	Natural killer cell mediated cytotoxicity	4
B cell receptor signaling pathway	4	Oxidative phosphorylation	4

At each time-point, the pathway classes were more than the results present in the table. Only these containing relative high gene numbers and low p-Value were chose for analysis.

### Validation of microarray data by qRT-PCR

**Figure 3:**
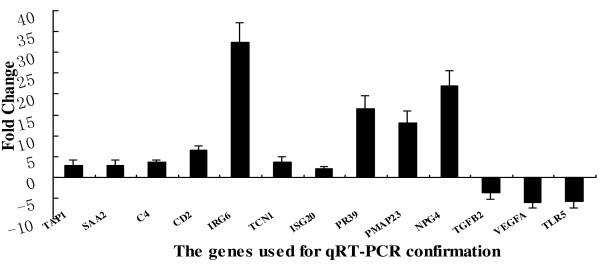
**Validation of the microarray data by qRT-PCR analyses**. The genes of *TAP1, SAA2, C4, CD2, IRG6, TCN1, ISG20, PR39, PMAP23 *and *NPG4 *were up regulated in SwIV infected pigs compared with normal controls; *TGFB2, VEGFA, TLR5 *were down regulated. Fold change is calculated based on the mean intensity value from 3 pigs by using the comparative Ct method. Significant levels were analyzed by T-test.

In order to get an overall validation of the microarray results, 13 genes with different levels of altered expression (10 up-regulated and 3 down-regulated) were selected for qRT-PCR confirmation, based on their involvement in different functional groups or pathways. Results showed that the altered expression of these 13 genes identified by microarray was consistent with the results of qRT-PCR (Figure 3), although the extent of the changes as measured by the two methods did not match exactly due to the different nature of the procedures (Table 4). What's more, qRT-PCR of 15 representative genes associated with anti-viral immune function were performed including TLRs and interferon-induced genes (ISGs) and found the similar tendency as microarray results (Table 5). The data could indicate that the results from the microarray analysis are good indicators of overall changes in gene expression.

**Table 4:** An overall validation of microarray data by qRT-PCR

Gene	GenBank ID	Microarray FC	qRT-PCR FC
TAP1	NM_001044581	2.46	2.83
SAA2	CB475095	8.42	2.86
C4	CF366280	2.95	3.53
CD2	NM_213776.1	2.93	6.43
IRG6	NM_213817.1	3.84	32.50
TCN1	BX675338	7.37	3.56
ISG20	NM_001005351.1	5.41	2.15
PR39	NM_214450	13.51	16.48
PMAP23	EW419136	21.7	13.07
NPG4	NM_213863	8.24	21.99
TGFB2	NM_214015.1	-2.55	-3.83
VEGFA	EU714324	-2.42	-6.20
TLR5	NM_001123202	-2.10	-5.78

Fold changes of gene expression in the porcine lung 3 days following H1N1 SwIV infection compared with the controls. The fold changes were calculated by the formula of 2^-delta-delta Ct^, data was means ± SD of triplicate reactions for each gene transcript. The expression level of *GAPDH *was assayed for normalization during quantitative PCR.

**Table 5:** A validation of Toll-like receptors and IFN-stimulated genes by qRT-PCR

Gene	GenBank ID	Microarray FC	qRT-PCR FC
TLR2	NM_213761.1	2.87	4.61
TLR3	NM_001097444.1	7.33	3.42
TLR8	NM_214187	2.68	2.44
TLR9	NM_213958	2.20	1.64
IRF3	NM_213770.1	4.32	6.33
IRF7	NM_001097428	3.25	11.1
Mx1	NM_214061	3.24	10.36
ISG15	EU584557.1	3.45	5.21
ISG20	NM_001005351.1	5.41	2.15
ISG54	XM_001928671.2	2.73	6.27
ISG56	XM_001928724;	3.39	3.43
ISG60	XM_001928703	2.47	5.70
GBP1	NM_001128473	2.78	12.0
OASL	NM_001031790	5.80	2.05
OAS2	NM_001031796	2.05	28.80

qRT-PCR results of 4 Toll-like receptors and 11 IFN-stimulated genes in the SwIV -infected pigs compared with the controls. Gene expression is depicted as relative expression compared to *GAPDH *gene expression.

## Discussion

Swine influenza is an acute respiratory disease caused by influenza A viruses that circulate among pigs with few or no clinical signs. However, after swine influenza infection, the altering host response predisposes to secondary bacterial infection in pigs through complicated mechanisms. At present, the molecular mechanisms of host factors involved in pathogenesis and secondary infection have not been clearly established due to the lack of overall information about host response after influenza infection in pigs. The pathogenesis of swine influenza resembles that of human influenza [25]. In humans, many studies have shown that the severe disease associated with influenza infection might arise through different mechanisms, and one important factor is the augmented host response induced by influenza virus [25]. In view of this, a genomic expression analysis was performed to obtain global information of the host response in lungs of infected pigs at different infection stages. While our clinical and pathological findings are in keeping with the findings of other authors, our unique immunological data allows for the ability to explain the short-lasting clinical signs and severe pathological changes over the course of illness through immune and inflammatory responses of pigs to H1N1 SwIV.

### Anti-viral innate immune response

**Figure 4:**
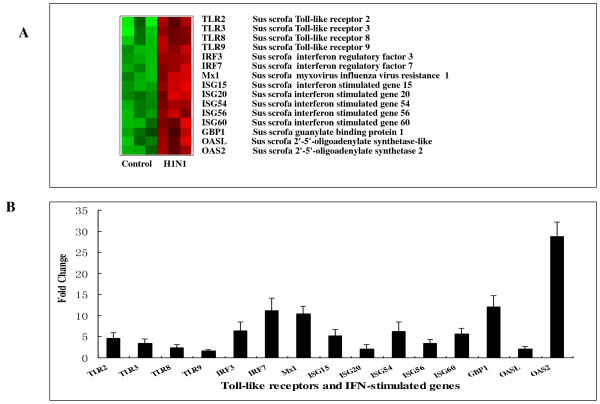
**The validation of Toll-like receptors and IFN-stimulated genes by qRT-PCR analyses**. (A) The genes in this bioset [differentially regulated by 2-fold or more (P < 0.05)] were clustered according to a hierarchical algorithm; red bars represent up-regulated levels of the 15 representative genes after H1N1 infection, including TLRs and interferon-induced genes (ISGs) which were involved in anti-viral signal molecular or effectors. (B) The genes of TLRs and interferon-induced genes (ISGs) were up-regulated at various degrees in H1N1 SwIV infected pigs compared with the normal controls.

The innate immune system functions as the first line of host defense against numerous pathogens. Pattern recognition receptors (PRRs) are a class of innate immune response expressed sensors that provide the host with the ability to detect and respond to virus infection, then trigger a class of anti-viral signaling cascades, ultimately ensuring production of necessary effectors molecules required for virus elimination, such as type-I interferons (IFNs). In this study, we found that several pattern recognition receptors were over-expressed in the infected pigs on PID 3, including intracellular sensors *(MDA5, RIG-I *and *TLR3,8,9*) and extracellular sensors (*CD14 *and *TLR2*) with differently increased folds, which was determined by qRT-PCR. With this in mind, we hypothesized that Toll-like receptors (TLRs) and RIG-I like receptors (RLRs) might be activated by SwIV then the receptors triggered the activation of NF-κB and IRF3/7, which cooperated in induction of antiviral type I IFNs [26]. Different TLR family detects distinct microbial pathogen associated molecular patterns (PAMPs) and triggers the activation of specific signaling pathways, leading to induction of interferons [27]. Though interferons were not observed up-regulated on PID 3, we hypothesized that interferons might be induced at the earlier time of infection, which could be indicated by the following enhanced production of ISGs [28]. ISGs are a large family of IFN-signaling and IFN-stimulated immune mediators, which may have a crucial role in antiviral response and host defense [29,30]. The present study found that on PID 3, ISGs particularly showed the increase of the expression to a relatively high level in response to H1N1 virus infection in pigs, and many showed even higher level, e.g. *OASL, OAS2, MX1, IRF7, IRF3, IFIT1, IFIT2, IFIT3, ISG15, ISG20 *and *ISG44*. Several recent microarray results have also highlighted the common involvement of IFN-mediated anti-viral responses in the acute phase of influenza infections in other animal models, and illustrated the role of interferons in the first line of defense against virus infections [31]. In addition, there are several functionally less well characterized interferon-induced genes, such as *INDO, CXCL9, CXCL10, GBP1, GBP2, GBP4, IFITM1 *and *CD274*. *CD274 *is up-regulated on lymphocytes upon IFN-gamma activation and plays a role in T cell co-stimulation and apoptosis during viral infections [32]. GBPs belong to the same families of IFN inducible GTPases as Mx1, and human *GBP1 *and *GBP2 *have been implicated in the resistance to VSV and ECMV infection [33]. The role of porcine GBPs in the influenza virus replication is in process of our research, but the experiment results achieved so far could imply that *GBP1 *has a significant anti-viral effect for influenza (unpublished data). The interferon-inducible transmembrane proteins (IFITMs) have recently been discovered to restrict an early replication of influenza A virus and flaviviruses, including dengue virus and West Nile virus [34]. The up-regulation of IFN-inducible genes after virus infection suggests that the expressions of these genes may play important roles in host antiviral activities. To further confirm the overrepresentation of antiviral genes, qRT-PCR of 15 representative genes was performed including TLRs and ISGs and the similar tendency as microarray results was observed (Figure 4). It was interesting to note that although the majority of up-regulated genes involved in the anti-viral immune response, three inhibitory genes were also up-regulated, including *ISG15, USP18 *and *ISG56*, which had been reported as the negative regulators of the RLRs signaling or IFN signaling [35-37]. This finding indicated that the production of these negative-regulated genes was an equally important factor of the host response which may keep extremely pulmonary response in check and help restore the host to its normal state when the infection is no longer present.

IFNs also stimulate the production of the MHC class I and II proteins which play important roles in the immune response to infections. In humans, *HLA-B *(major histocompatibility complex (MHC); class I; B) and *HLA-DRB1 *(MHC; class II; DR beta 1) are involved in antigen presentation and the connection of the innate immune system with the adaptive immune system. In the present study, interestingly, both of the MHC genes, *SLA-B *and *SLA-DQA *were up-regulated, at 3.2-fold and 5.2-fold respectively. Previous studies have demonstrated that viruses have evolved mechanisms to inhibit MHC class I expression by interfering with the function of the MHC class I assembly machinery in the endoplasmic reticulum and by exploiting endoplasmic-reticulum-associated degradation pathways [38]. Different influenza strains demonstrate various abilities in modulating the mRNA expression of MHC class. On the one hand, H3N2 influenza virus could cause the up-regulation of MHC class I mRNA expression levels [39]. On the other hand, the expression of MHC class I did not increase due to the infection of macrophages with a low pathogenic H7N2 AIV [40]. Thus, it can be suggested that H1N1 SwIV may be unable to inhibit MHC genes expression, which should be an important host factor for easily controlling virus.

Besides the above anti-viral agents, another important defense component of an animal innate immune response is constitutive or inducible production of antimicrobial host defense peptides (HDPs). Besides MAPK and JAK/STAT signaling pathways, interaction between virus and TLRs can trigger the *NF-κB *transcription factor, thus activating down-stream signaling pathway responsible for producing HDPs [41]. In this study, we detected several high-expression antimicrobial peptides on PID 3, such as *PMAP23, PR39, CAMP *and *NPG4 *which were all up-regulated with 21.7-fold, 13.5-fold, 8.3-fold and 8.2-fold respectively. A correlation between the acute SwIV infection and the high level of HDP production in pig lungs was demonstrated by qRT-PCR. To evaluate antiviral activity of the HDPs, *PMAP23 *and *NPG4 *were chosen to further characterize the function in vitro. The primary results demonstrated that in most cases, either peptides pre-incubation with virus prior to addition to cells, or simultaneous addition to cells in cell culture medium, yielded similar results with regard to inactivation of viral infectivity in a dose-dependent manner (unpublished data). These findings suggest that HDPs contribute to an early host defense against SIV infection which may be the potential antiviral effectors of pigs.

### Inflammatory responses

When successfully overcoming the first barrier of host defense, influenza virus will infect the lung epithelial cells and macrophages then activates the production of pro-inflammatory cytokines and chemokines through distinct signaling pathways [42,43]. Using DNA microarrays, the gene expression patterns that correlate with inflammatory response of virus infection can be identified. Through such approach, it has been reported that different animals (such as primates, ferrets and mice) with influenza infection have been induced extraordinarily high expression of cytokines and chemokines (e.g. *CXCL10, CCL2 *and *CXCL9*) [44-46]. In the current report, we found that a large amount of genes involved in inflammatory response were up-regulated at various degrees after H1N1 SwIV infection, such as *SERPINA3, CXCL9, CXCL10, CD163, CD14, CCR5, CCL26, C4 *and *ALOX15*. Furthermore, we also found that thirteen genes involved in Toll-like receptors pathway and another thirteen genes in MAPK signaling pathway were regulated, which reflected the up-stream signaling cascades that could lead to secretion of inflammatory cytokines and chemokines. The role of pro-inflammatory cytokines and chemokines in the pathogenesis of H1N1 virus infection in mammal remains controversial. For instance, in response to pro-inflammatory cytokines, immune cells migrate toward the areas of infection where they exert host defense functions by phagocytosing cell debris and invading microorganisms, and eventually controlled the infection. However, the overabundant productions of inflammatory responses are responsible for the signs and pathogenesis of influenza infection [47,48]. For example, *CXCL10 *is thought to play a role in the temporal development of innate and adaptive immunity in concert with type I and II IFNs [49]. However, previous studies *in vivo *have also shown that *CXCL10 *potentially contributes to lung pathology of H1N1 virus infection [50]. In addition, the receptors of chemokines play an important role in site-directed migration and activation of leukocytes. In our studies, *CCR5, CCR10 *and *CXCR6 *were slightly up-regulated at the early infection. Previous studies have proved that avian H1N1 influenza virus could enhance expression of *CCR5 *in infected adult MDM**s **[51]. Using knock-down mice model, *CCR5 *was demonstrated to play a significant role in reducing the mortality rates of mice after influenza virus infection, which indicated that *CCR5 *significantly affected the course of immune mechanisms as well as the clinical outcome, although in a profound way [52]. In addition, it has been deeply studied that cell adhesion molecules, which can mediate virus-elicited alveolar monocyte accumulation in SwIV-infected pigs, play essential roles in many immune and inflammatory responses [53]. In the current study, through KEGG pathway analysis, we found that cell adhesion molecules (CAMs) pathway was the significant one during the acute virus infection, which contained the families of immunoglobulin superfamily (*IGSF21*), selectins (*SELL, SELE*), and others like *F11R, CD274, CD2, CR2 *and their ligands. Briefly, the above results suggest that the production of inflammatory cytokines and cell adhesion molecules in pigs could not only contribute to the control of virus replication but also elicit significant tissue damage of lung in the context of acute H1N1 virus infection.

In order to keep homeostasis of development and metabolism, the host must express anti-inflammatory factors to prevent excessive inflammatory damage caused by infection. In this study, we found several genes were regulated after influenza infection which involved in anti-inflammatory functions through PPAR signaling pathway especially on PID 7. The activation of PPAR pathway can play the significant role in anti-inflammatory process because they reduce expression of several pro-inflammatory cytokines, chemokines, and cell adhesion molecules [54]. Except for genes of PPAR signaling, we also detected up-regulation of other individual anti-inflammatory factors, such as *IL1-RA, IL2RA, HSP40 *and *HSP70*. Interestingly, as the anti-inflammatory mediator, *TGF-β2 *was found to be down-regulated for SwIV infection throughout the whole process, which was demonstrated by qRT-PCR. Previous studies showed that human H1N1 virus could induce a persistent elevation of *TGF-β2 *mRNA at human pulmonary epithelial cells [55], while highly pathogenic H5N1 virus could cause a down-regulation of *TGF-β *secretion in mice model which resulted in more severe and widespread lesions [56]. Whether the down-regulation of TGF-β was caused by swine influenza or responsible for acute lung immunopathology of early infection of swine influenza remains to be further studied. From these analyses, we could propose that the infection course is the balance of pro-inflammatory versus anti-inflammatory factors, and these in common affects clinical outcome of influenza virus infections.

### Acute-phase response

Acute phase proteins (APPs), such as *LTF, SAA2 *and coagulation factor XIII, were involved in physiologic reactions initiated early in the inflammatory process [57]. Acute phase proteins were also reported as host antiviral factors against virus infection. In response to influenza virus infection, numerous acute phase proteins in plasma, such as *CRP, SAA1*, and *Orosomucoid*, increased dramatically in concentration [58,59]. In the present study, we found several genes such as *SERPINA3, MBL2, F2, CD16*3, *SAA2 *and lactotransferrin were all up-regulated with 3.12-fold, 2.57-fold, 2.14-fold, 2.63-fold, 8.43-fold and 12.4-fold respectively on PID 3. These up-regulated genes are representative of acute response of host and may help host eliminate the invading viruses. However, it is interesting to note that the genes including Alpha-2-macroglobulin (*A2M*), *Transferrin *and *Fibronectin *were down-regulated on PID 3. *A2M *has been identified as the inhibitor of influenza A virus in pig serum with virus-neutralizing activity [60]. Recently, using proteomics method, researchers identified *A2M *in the salivary of people infected with swine origin influenza A virus (S-OIV), and explored *A2M *as a novel receptor-targeted inhibitor against S-OIV [61]. The cause and effect of down-regulated expression of these genes associated with acute-phase response need further study.

### The expression of the immune cell markers

Cellular immune responses are critical for the complete clearance of the influenza virus [62]. Pigs infected with H3N2 and H1N1 viruses have an increased frequency of neutrophils, NK cells, and CD4 and CD8 T cells in the BAL fluid [63]. As pulmonary inflammation after influenza virus infection often involves immune cells infiltration, it raises the question whether the host response occurs primarily in sessile lung cells or could be attributed to infiltrating immune cells. In the current study, several genes as immune cell markers were also detected moderately up-regulated, containing some markers associated with monocytes and macrophages (e.g. *CD14, CD163*) or T lymphocytes (e.g. *CD2, CD3*ε and *CD8A*) on PID 3 [64]. In some cases these CD molecules do show a parallel expression pattern, as expected if the gene expression responses are caused by cellular influx. Recently, some researchers have found that higher frequencies of cytotoxic T lymphocytes, dendritic cells, activated T cells, and CD4+ and CD8+ T cells could be detected in SwIV-infected pig lungs by flow cytometric analysis [13]. In addition, leukocyte/endothelial cell adhesion molecules are essential mediators of both immune and inflammatory responses, especially implicated in leukocyte recruitment, trafficking and tissue damage [52]. In the current study, some cell adhesion molecules were detected over-expression during the acute virus infection, such as the families of immunoglobulin superfamily (*IGSF21*), selectins (*SELL, SELE*), and other molecules like *CD274, CR2 *and their ligands, which could be considered as the indirect evidences for leukocyte recruitments. Therefore, these suggested that the host immune and inflammatory responses in our study could be partly attributed to immune-related cells influx after virus infection. Other functional classes of DE genes involved in host response might be essentially dependent on gene expression changes in sessile pulmonary cells. Further study is suggested to be focused on the impact of immune cells influx to transcriptional changes of lungs after SwIV infection.

## Conclusions

In this work, we compared host clinical signs and pathology changes between infected pigs with H1N1 SwIV and the controls at the different stages, and subsequently determined 268 DE genes on PID 3 and 214 DE genes on PID 7 respectively, through DNA microarray analysis under the specific criteria. Our data explained that a series of genes involved in host defense responses were activated after the H1N1 SwIV invasion, which might be contributors to eliminating virus and severe immunopathology associated with virus infections. The comparison of the overall pattern of antiviral signaling and pro-inflammatory pathways activated by SwIV, it could help to screen the potential host agents for reducing the prevalence of SwIV and further understand the pathogenesis of swine influenza infection in pigs.

## Abbreviations

SwIV: swine influenza virus; PID: day post infection; DE: differential expression; FC: fold change; GO: Gene Ontology; KEGG: Kyoto Encyclopedia of Genes and Genomes; FDR: false discovery rate; qRT-PCR: quantitative real-time PCR; TLR: Toll-like receptors; HDPs: host defense peptides.

## Competing interests

The authors declare that they have no competing interests.

## Authors' contributions

YTL and HBZ carried out all works in the lab and drafted the manuscript. ZBW, SJW and HCC made substantial contributions to bioinformatics and statistical analysis. CHH and GMJ participated in the animal challenge experiment. MLJ conceived the study and participated in its coordination and helped to draft the manuscript. All authors read and approved the final manuscript.

## Supplementary Material

Additional file 1 **Primers used for qRT-PCR validation**. Click here for file

Additional file 2 **The overlapping DE genes on PID 3 and 7**. Click here for file

Additional file 3 **The complete microarray dataset of DE genes on PID 3 and 7 with functional annotations and expression level changes**. Click here for file
