# Supplementary material for: Transcription analysis on response of swine lung to H1N1 swine influenza virus
Source: BMC Genomics. 2011 Aug 8;12:398. doi: 10.1186/1471-2164-12-398 (PMC3169531; doi:10.1186/1471-2164-12-398)
Supplement: Additional file 1 — Primers used for qRT-PCR validation. [file 1471-2164-12-398-S1.DOC]

## Primers used for QPCR validation and additional expression profiling.

| Gene name | Forward primer | Reverse primer |
| --- | --- | --- |
| TLR2 | TCACTTGTCTAACTTATCATCCTCTTG | TCAGCGAAGGTGTCATTATTGC |
| TLR3 | AGTAAATGAATCACCCTGCCTAGCA | GCCGTTGACAAAACACATAAGGACT |
| TLR8 | AAGACCACCACCAACTTAGCC | GACCCTCAGATTCTCATCCATCC |
| TLR9 | CACGACAGCCGAATAGCAC | GGGAACAGGGAGCAGAGC |
| IRF3 | GTGGTGCCTACACTCCTG G | CTGTGGTCCTCTGCTAAACG |
| IRF7 | CCCACTGACCCTCATAAGG | CAGCCTCTCACCAGTATGTG |
| Mx1 | AGCGCAGTGACACCAGCGAC | GCCCGGTTCAGCCTGGGAAC |
| GBP1 | AGCACCTTCGTCTACAACAGC | TCAGCCGAGTCCTCAATCC |
| ISG15 | GATCGGTGTGCCTGCCTTC | CGTTGCTGCGACCCTTGT |
| ISG54 | ACGCAGAAGACGGCAGAGA | AGGCAGGCGAGATAGGAGC |
| ISG56 | CTGGGTATGCAATCACCATCTATCG | TCTTCGGTAAAACTTGACTGCGTGT |
| ISG60 | GCATTTTCCAGCCAGCATCT | TTCCTGTCTCTGTCAGCCCC |
| OAS1 | GACCTCGTCGTCTTCCTCACC | TTCTCCCGTCGTGGACTCTG |
| OAS2 | CACAGCTCAGGGATTTCAGA | TCCAACGACAGGGTTTGTAA |
| PR39 | GAACCCATCCAATGACCCAC | TGTTATCAGCCACTCCATCACC |
| PMAP23 | GCTGGTCACTGTGGCTTCTG | TCCGAGGACTGCTCGTTGA |
| NPG4 | CTGGTCACTGTGGCTTCTGC | AGATTAGCTTCCGAGGACTGC |
| SAA2 | CGCCTGGGCTGCTAAAGT | CACGAGGTCTGAAGTGGTTGG |
| C4 | ACCAAGCGACACCTCATACCT | GCAGAAACCTTGACGGGAAT |
| CD2 | AAAGCAGAGCAGCAGGAGAA | AGGAGTTTGGGAAATGACCG |
| IRG6 | CGTGTCCTGCTTGGTGCC | GATCCTTCCGCCCGTTTC |
| TCN1 | ATGAAGGCGGCTCAGGAA | CCACGGAGGAGGGTTTGA |
| TAP1 | GTGTGCGACGGAATCTATAAC | GAGCATGAGCCCTAAGAGAC |
| ISG20 | TACAGAACACGGGTCAGCG | TTTCAGTGCCTGGAAGTCG |
| TGFB2 | GCTGCTGTGGCTGCTAGTG | TCGCGGGTACTGTTGTAAAG |
| TLR5 | CTCGCCCACCACATTA | TGAGGGTCCCAAAGAGT |
| VEGFA | TTCCAGGAGTACCCCGATGA | TGGCCTTGGTGAGGTTTGAT |
| GAPDH | TGCCAACGTGTCGGTTGT | TGTCATCATATTTGGCAGGTTT |
