# Supplementary material for: Transcription analysis on response of swine lung to H1N1 swine influenza virus
Source: BMC Genomics. 2011 Aug 8;12:398. doi: 10.1186/1471-2164-12-398 (PMC3169531; doi:10.1186/1471-2164-12-398)
Supplement: Additional file 2 — The overlapping DE genes on PID 3 and 7. [file 1471-2164-12-398-S2.DOC]

## The overlapping DE genes on PID 3 and 7.

| **Gene description** | **Gene symbol** | **Fold Change（PID 3）** | **Fold Change（PID 7）** |
| --- | --- | --- | --- |
| Complement component C8A | C8A | 2.5177433 | -3.245079 |
| Sus scrofa chemokine ligand 26-like mRNA | CCL26 | 8.645769 | 2.0751038 |
| *Rep:CD274 molecule CD274, mRNA- Homo sapiens* | CD274 | 2.648947 | -2.200339 |
| Sus scrofa immunoglobulin kappa variable group | CNGA1 | -2.832141 | -9.567118 |
| cytochrome c oxidase subunit 8C | COX8C | 16.659502 | 11.793154 |
| Complement receptor type 2 | CR2 | 3.357186 | 2.0073078 |
| Sus scrofa exosome component 10-like | EXOSC10 | -4.2016826 | -4.4323087 |
| Sus scrofa ficolin collagen FCN1 | FCN1 | 3.0437126 | 2.7339246 |
| DnaJ Hsp40 homolog, subfamily B, member 9 | HSP40 | 2.1262078 | -2.287076 |
| indoleamine 2,3-dioxygenase 1 | IDO1 | 2.253654 | -2.6277351 |
| Interferon-stimulated gene 20 kDa protein | ISG20 | 5.414398 | 2.1768405 |
| Sus scrofa lymphocyte-activation gene 3 | LAG3 | 4.2203608 | 2.0111256 |
| Sus scrofa lactotransferrin | LTF | 12.400818 | 2.4587727 |
| Sus scrofa matrix metallopeptidase 13 collagenase 3 | MMP13 | - 2.2246888 | 2.3383074 |
| Sus scrofa peroxisome proliferator-activated receptor delta gene | PPARD | 3.0221992 | - 2.4203787 |
| Sus scrofa STEAP family member 4 | STEAP4 | 2.484801 | 2.0804205 |
| Sus scrofa haptocorrin 1 | TCN1 | 7.374038 | 6.530724 |
| *Rep：Macaca mulatta transforming growth factor, beta 2* | TGFB2 | - 2.5542269 | - 2.180843 |
| similar to tranglutaminase 1 | TGM1 | 4.191665 | 3.2574801 |
| Sus scrofa Toll-like receptor 3 | TLR3 | 7.3319163 | - 2.5434992 |
| Sus scrofa tumor necrosis factor superfamily member 18 | TNFSF18 | 2.0651836 | 2.5221772 |
| Sus scrofa vascular endothelial growth factor A | VEGFA | - 2.422289 | - 2.3052964 |
| *Rep：WNT inhibitory factor 1* | WIF1 | - 3.179808 | - 2.5332527 |
